# Efficient search for a face by chimpanzees (*Pan troglodytes*)

**DOI:** 10.1038/srep11437

**Published:** 2015-07-16

**Authors:** Masaki Tomonaga, Tomoko Imura

**Affiliations:** 1 Primate Research Institute, Kyoto University, 41-2 Kanrin, Inuyama, Aichi 484-8506, JAPAN; 2 Niigata University of International and Information Studies, 3-1-1 Mizukino, Nishi-ku, Niigata, Niigata 950-2292, JAPAN

## Abstract

The face is quite an important stimulus category for human and nonhuman primates in their social lives. Recent advances in comparative-cognitive research clearly indicate that chimpanzees and humans process faces in a special manner; that is, using holistic or configural processing. Both species exhibit the face-inversion effect in which the inverted presentation of a face deteriorates their perception and recognition. Furthermore, recent studies have shown that humans detect human faces among non-facial objects rapidly. We report that chimpanzees detected chimpanzee faces among non-facial objects quite efficiently. This efficient search was not limited to own-species faces. They also found human adult and baby faces-but not monkey faces-efficiently. Additional testing showed that a front-view face was more readily detected than a profile, suggesting the important role of eye-to-eye contact. Chimpanzees also detected a photograph of a banana as efficiently as a face, but a further examination clearly indicated that the banana was detected mainly due to a low-level feature (i.e., color). Efficient face detection was hampered by an inverted presentation, suggesting that configural processing of faces is a critical element of efficient face detection in both species. This conclusion was supported by a simple simulation experiment using the saliency model.

The face is quite an important stimulus for humans. Faces convey various kinds of information that are critical for our social lives. Many studies have shown that faces are processed rather differently from other object categories. For example, we are severely hampered^1^,^2^ when we see upside-down faces. Furthermore, when a first-order spatial configuration among facial features (horizontally arranged eyes and nose and mouth located beneath the eyes) is distorted, face recognition is readily disturbed^3^. This result strongly suggests that humans process faces in a holistic manner. This type of processing is most evident for faces, although expertise (long-term intensive experience) on specific object categories also causes a shift in processing from featural to holistic processing^4^,^5^.

Faces also capture our visual attention quickly. For example, when a face is presented abruptly in peripheral vision, our visual attention shifts automatically^6^,^7^,^8^. Attention to a face can be examined in terms of the presence of “pop-out” or “efficient” searching when using a visual search paradigm^9^,^10^. When a search display contains only facial stimuli and observers are required to find a specific face among other faces, researchers often fail to find pop-out effects^11^. In contrast, when a face appears among non-face objects (such as cars, houses, etc.), it is detected without any “effort”. Hershler and Hochstein clearly demonstrated this phenomenon using various stimuli and experimental conditions^12^. They suggested that the pop-out effect or very efficient searching is caused not only by low-level “features”, as Treisman *et al.* initially proposed^9^, but also by higher-order object categories such as faces^12^,^13^, although controversies remain^14^,^15^.

Face perception in nonhuman primates has also been intensively studied from the cognitive-neuroscientific and comparative-cognitive perspectives^1^,^16^,^17^,^18^,^19^,^20^,^21^,^22^,^23^,^24^,^25^,^26^,^27^,^28^,^29^. In particular, there is accumulating evidence of various aspects of perception and cognition related to faces in chimpanzees: the face-inversion effect^16^,^18^,^21^,^22^, perception of facial expression^30^,^31^, hemispheric specialization of face processing^19^,^21^, perception of gaze perception^23^,^32^,^33^, perception of a Mooney face^34^, development of facial recognition^20^,^35^, and effects of long-term experience^5^. These findings suggest that chimpanzees perceive faces in a holistic manner, as do humans. Furthermore, chimpanzees exhibit results similar to humans regarding spatial attention caused by facial stimuli^8^. These findings suggest that chimpanzees also rapidly detect a face among non-face objects, as determined in humans by Hershler and Hochstein^12^.

In the present series of experiments, we examined efficient searching for faces by three adult chimpanzees using visual search tasks^9^,^10^,^36^,^37^,^38^ (Fig. 1). In the first experiment, the chimpanzees were trained to find a picture of a chimpanzee face, a banana, a car, and a house among distractors from various object categories (Fig. 2). All target categories were familiar to our chimpanzee participants, but we predicted that only a face would be detected efficiently. In the second experiment, we manipulated low-level and higher-order features to identify the contributions of these features, particularly the role of first-order spatial configuration of the face. To confirm the results of the second experiment, we conducted simple simulation experiments using the saliency-map model^39^,^40^. This model simulates attention based on a set of low-level features, such as colors, contrasts, line orientations, etc. In the last two experiments, we examined the generality and limitations of efficient searching for facial stimuli among non-face objects by presenting other-species faces as well as profiles and outer features of faces.

Note that some parts of the experiments reported here were very briefly and preliminarily described in one section of a book chapter by the first author^41^, but only the results summary was presented and not the detailed descriptions of methods or the statistical analyses conducted. In this study, we fully describe the methods of, and results from, those additional experiments.

## Results

### Experiment 1: Efficient searching for a chimpanzee face

Three adult female chimpanzees, Chloe, Pendesa, and Ai, participated in the experiments. They lived together with 14 chimpanzees (including themselves) in an environmentally enriched outdoor compound^42^. They had participated in various perceptual and cognitive experiments, including visual searching^22^,^23^,^36^,^37^,^38^,^43^,^44^. After their initial training (see Supplementary Fig. S1), the chimpanzees underwent test sessions. One type of target (chimpanzee face, banana, car, or house) appeared in each session. The set size (i.e., number of stimuli in the search display) varied among 5, 10, and 18 from trial to trial. The trial in which the homogeneous distractors appeared and that in which distractors were heterogeneous alternated between sessions (Fig. 2).

Figure 3 shows the results of the heterogeneous-distractor trials (see Supplementary Fig. S3 for the homogeneous distractor results). The chimpanzees showed very high accuracy for the chimpanzee facial target in contrast with the car and house targets (general linear mixed-model analysis, F(3,187) = 68.94, P = 4.55 × 10^−30^). This was also true for the response-time data (F(3,204) = 83.42, P = 2.94 × 10^−35^). Furthermore, interactions between set size and target were also significant for accuracy (F(6,187) = 4.57, P = 2.36 × 10^−4^) and response times (F(6,204) = 5.92, P = 1.02 × 10^−5^). Chimpanzees detected the conspecific face more efficiently than the car or house targets. Interestingly, they also accurately and rapidly detected a photograph of the banana. Although accuracy for the chimpanzee target was significantly better than that for the banana target (multiple comparison based on unweighted means, P = 0.045), no significant difference in response times was observed between these two targets.

### Experiment 2: Stimulus manipulation test

The first experiment showed that the chimpanzees detected a chimpanzee face accurately and quickly. However, such efficient search was also observed when they searched for a banana. Thus, the next question to be addressed was whether the chimpanzees searched for these targets in the same manner. If higher-order properties caused efficient searching for a face, manipulations that deteriorate those properties would severely hamper search performance. In contrast, if the chimpanzees used simpler, low-level features, such as color, during the search for the banana, manipulations that change such properties would affect search performance. In the second experiment, the chimpanzee face and banana targets were manipulated in three ways, such as inversion (rotated 180°), grayscale (color information removed), and scramble (edge information removed) (Fig. 4).

Figure 4 shows the mean response times for the correct trials. A significant interaction was detected between target type and stimulus manipulation (F(3,140) = 25.60, P = 2.91 × 10^−13^). Chimpanzees showed significantly longer response times when the face was inverted and scrambled than when it was intact (multiple comparisons, upright vs. inverted, P = 0.002; upright vs. scrambled, P = 8.22 × 10^−12^), whereas the grayscale had no significant effect. In contrast, when the grayscale picture of the banana was presented as the target, response times were significantly longer than those during baseline intact target trials (multiple comparison, upright vs. grayscale, P = 1.33 × 10^−9^). Inversion and scrambling had no effect on search performance. These results clearly indicate that the chimpanzees searched for the face and banana in a different manner. Higher-order features, such as first-order spatial configuration, was one of the sources for efficient searching for faces, whereas the low-level feature (color) caused efficient searching for the banana. The accuracy results were similar to those for the response times and are described in Supplementary Materials.

### Experiment 3: Simulation with the saliency map model

To confirm the results of the first experiment, we conducted a simple simulation experiment. We used the “saliency map model” for this simulation^39^,^40^. This model is derived from cognitive and neurophysiological evidence on object recognition. This model localizes the salient areas based on low-level features, such as color, brightness, and line orientation (also curvature), and the most salient area captures the observer’s attention using a “winner-take-all” principle. This model simulates how spatial attention is guided in a bottom-up manner. We ran 2,160 heterogeneous-distractor trials using this model, which was randomly generated by a computer, to yield simulated accuracy data. If the model detected the predefined target within scans less than or equal to the set size, the trial was defined as “correct”. Figure 5 shows the error percentage results for Experiment 1 and the current simulation experiment. The results from these two experiments were similar, with the exception of those for the chimpanzee face. A general linear mixed-model analysis conducted on the simulation data showed a significant main effect of target type (F(3,187) = 73.19, P = 2.35 × 10^−31^), and the facial search was most difficult in the saliency model (multiple comparisons, Ps < 0.001). This result was completely opposite from the actual chimpanzees’ performances. These results strongly indicate that visual searching for a face is qualitatively different from searching for other object categories. Chimpanzees and humans utilized non-low-level features to search for a face.

### Experiment 4: Visual search for other-species face

In the next two experiments, we examined the generality and limits of efficient searching for a face. In previous experiments, we used the chimpanzee face as the target. In this experiment, we presented the faces of human females (Caucasian), as well as those of babies, Japanese macaques, and chimpanzees. The participant chimpanzees were very familiar with Caucasian females (as experimenters) and macaques (living in adjacent enclosures) but had almost no experience with human babies. Thus, we expected that the chimpanzees would detect the human female and macaque faces more efficiently than the human baby faces if familiarity had some effect on searching efficiently for a face. However, as shown in Fig. 6, the chimpanzees rapidly detected the human face, regardless of age, and showed significantly slower response times for the macaque facial target (F(3,33) = 35.70, P = 1.80 × 10^−10^; multiple comparison, macaque face vs. others, Ps < 0.001). The accuracy results were similar to those for the response times and are described in Supplementary Materials.

### Experiment 5: Visual search for a profile face and outer features

The final experiment examined the effect of other facial properties while efficiently searching for a face. One was the effect of gaze direction and the other was inner/outer features. A mutual gaze captures a human observer’s attention^6^,^7^. Humans show efficient searching for a direct-gaze face more than an averted-gaze^45^,^46^. This is also true of chimpanzees^8^,^23^. If such an eye property plays some role during visual searching for faces, chimpanzees would be more efficient when searching for a direct-gaze face than an averted-gaze face. Thus, we prepared front-view faces and profiles of chimpanzees and human females. The left and center panels of Fig. 7 show the mean response times for correct trials during the visual search for front-view vs. profile faces of chimpanzees and humans. The chimpanzees exhibited efficient searching for front-view faces compared to that for the profile-view faces under both conditions (chimpanzee face, F(1,100) = 96.03, P = 2.73 × 10^−16^; human face, F(1,85) = 105.15, P = 1.58 × 10^−16^). Although the effect of set size was significant under both conditions (chimpanzee face, F(2,100) = 43.04, P = 3.28 × 10^−14^; human face, F(2,85) = 23.14, P = 9.51 × 10^−9^), the two-way interactions did not reach significance (chimpanzee face, F(2,100) = 1.80, P = 0.171; human face, F(2,85) = 2.950, P = 0.058). The accuracy results were similar to those for the response times and are described in Supplementary Materials.

In the second series of tests, we examined the effects of outer/inner features of faces^12^,^47^. According to the stimulus manipulation test results, efficient searching for faces was suggested to be derived from configural processing of faces. If this was true, the chimpanzees would show deteriorated performance when configural features; i.e., inner features, were masked. To test this possibility, we presented two types of chimpanzee faces: one was an intact face, as in previous conditions, and the other was a face in which the inner features were masked (Fig. 7). As shown in the right panel of Fig. 7, the chimpanzees showed worse performances on masked faces than that on intact faces (stimulus type, F(1, 85) = 79.39, P = 8.21 × 10^−14^; set size, F(2,85) = 42.32, P = 1.76 × 10^−13^; interaction, F(2,85) = 4.16, P = 0.019). These results further confirm the contribution of configural processing to efficient searching for faces.

### Learning effect

In the present experiments, the same three chimpanzees were exposed to all conditions successively; thus, they were repeatedly exposed to a search display of an upright chimpanzee face. Such repeated presentations of a chimpanzee face would have caused an over-learning effect. Then, the results of response times in the later series of tests could have been explained by the over-learning effect: upright faces were detected faster than other types of targets simply because they were presented repeatedly. To rule out this possibility, response times for upright front-view chimpanzee faces were compared across experimental conditions. Chimpanzees showed no difference in response times across experimental conditions. The results of one-way ANOVA using the response time data at the set size of 10 under five conditions with upright chimpanzee faces conducted in four experiments indicated a nonsignificant main effect of condition (F(4,8) = 3.13, P = 0.08).

## Discussion

We examined how chimpanzees efficiently search for faces among non-face objects. In the first experiment, we found that chimpanzees detected a chimpanzee face among non-face distractors quite quickly and readily. Although they also rapidly detected a banana, this was simply because they paid attention to the color, as shown in Experiment 2. The search slope for each target was 19 ms/item (standard error [SE] = 8 ms) for the chimpanzee face, 26 ms/item (SE = 4 ms) for banana, 62 ms/item (SE = 9 ms) for car, and 52 ms/item (SE = 25 ms) for house. The search slope for faces was considered an “efficient search”^10^,^36^, although the value was apparently larger than that from the human experiment (6 ms/item)^12^. This may have been due to various procedural differences, such as response types, set size, and so on.

In contrast with the upright presentation, their performances were hampered significantly when the chimpanzee face was inverted, but this manipulation had no effect on detecting the banana (Experiment 2). These results clearly suggest that holistic or configural processing of faces played an important role during efficient searches for faces^16^,^18^,^21^,^22^. This conclusion was further supported by a simple simulation experiment using the saliency map model^39^,^40^. The simulation results were similar to the actual results of chimpanzees when the targets were the banana, house, and car, suggesting that the searches for these targets by chimpanzees were mainly guided by low-level salient features. However, the simulation results with chimpanzee-face targets were completely different from those of chimpanzees; searching for a face in the simulation program was worse than the other target types (Experiment 3). These results clearly indicate that the spatial configuration or a combination of facial features, not simply a feature, is more responsible for efficient searching for faces. These results are consistent with the reverse hierarchy theory of Hochstein and Ahissar^13^, and consistent with our previous findings of attentional capture of faces by chimpanzees^8^. As in humans, the attention of a chimpanzee is readily captured by a face.

In subsequent experiments, we further investigated the extent and limits of efficient searching for faces in chimpanzees. Chimpanzees exhibited efficient searching for faces of adult and baby humans, but not for monkey faces (Experiment 4). These results were surprising because the amount of visual experience for monkey faces is much larger than that for human babies. Actually, the current chimpanzee participants had no visual contact with human babies. Some other additional properties concerning facial processing may have affected our results. One possibility is the bias in low-level saliency among stimuli used in this experiment. Therefore, we conducted additional saliency map model simulations using the same stimuli. As a result, the percentage of “error” for monkey faces was significantly higher (41.7%) than for the two types of human faces (baby: 20%, adult female: 19.4%, general linear mixed-model analysis, Ps < 0.001) and was comparable to the chimpanzee faces (40%). It may be that the brighter skin of the human faces enhanced the contrast between the facial features and the background, or that the reddish skin of the monkey faces weakened this contrast. To examine the effects of low-level features on the present results, we should conduct the same types of stimulus manipulation tests for these stimuli as in Experiment 2. In Experiment 2, we manipulated the stimuli by reducing the color properties, by rotating 180 degrees, etc. These manipulations may shed light on contributions of simple and more configural features in the current results. Unfortunately, we did not conduct these tests, but this possibility should be examined in the future. The other possibility is familiarity based on long-lasting social experiences^5^. In humans (and presumably chimpanzees), perceptual narrowing for face perception occurs during infancy. A 9-month-old human infant is more sensitive to conspecific faces^48^. This perceptual narrowing is considered a mechanism for the own-race face effect^49^. Furthermore, recent studies with chimpanzees strongly suggest that long-lasting experiences and modified facial processing continue into adulthood^5^. Although facial patterns including conspecific faces, other-species faces, and schematic faces are processed holistically if they contain first-order configurations of facial features^22^, efficient searching for faces requires familiarity established through long-lasting social interactions. Additionally, animal faces do not cause efficient searching in humans^12^, possibly due to the same reasons as in chimpanzees. Our chimpanzees efficiently searched for human baby faces, with which they had no visual contact. However, the human baby face was more similar to an adult human face than a monkey face. This high similarity explains the current results.

The efficiency of searching for faces decreased after removing the inner features of the faces (Experiment 5). This result also supports the importance of holistic processing of faces while efficiently searching for faces. Interestingly, humans efficiently search for faces using with inner or outer features, although searching for inner-features of a face is more efficient than searching for outer features^12^. The reason for this discrepancy between species is unclear. Configural properties are more evident for inner features than outer features. Our results suggest the extent and limits of holistic processing of faces by chimpanzees.

Efficient searching was also limited to a front-view face (Experiment 5). When we presented the profile-view face, chimpanzees exhibited significantly prolonged search times than those for a front-view face. This result cannot be explained by prolonged exposure (leading to overlearning) to the front-view face target trials. No significant differences in the response times to upright front-view chimpanzee faces were observed across experiments. A direct-gaze face in humans is more efficiently searched than an averted-gaze face^45^,^46^. This is also true in chimpanzees^23^. In addition, humans can detect a face with an angry expression more rapidly than a neutral face^50^,^51^. Therefore, attentional capture by the face can be modulated by various facial features, particularly social-communicative features. Our results suggest that visual searching for faces by chimpanzees is also affected by these social-cognitive factors. A front-view face contains eyes with a direct gaze. This social-attentional property may have affected our results.

Chimpanzees efficiently searched for facial stimuli as humans did. This efficient searching was hampered by an inverted presentation. The present results provide further evidence for holistic or configural processing of faces by chimpanzees. As noted, faces are quite important for social interactions. When comparing humans and chimpanzees, there are some differences in the style of social interactions between them, especially in the joint-attention contexts^52^. On the basis of the current findings, we can examine further the origin of different social interactions in chimpanzees and humans.

## Methods

### Participants

Three adult female chimpanzees (*Pan troglodytes*)—Chloe, Pendesa, and Ai—participated in the present experiments. Chloe was 25, Pendesa was 28, and Ai was 29 years of age at the onset of the experiments. All participants lived in a social group of 14 individuals (including themselves) in an indoor and environmentally enriched outdoor compound (770 m^2^) at the Primate Research Institute, Kyoto University (KUPRI), Japan^42^. They were fed fruits, vegetables, and primate chow three times per day. The chimpanzees had engaged previously in various computer-controlled perceptual and cognitive tasks, including visual search tasks^22^,^23^,^36^,^37^,^38^,^43^,^44^.

### Ethics Statements

The care and use of the chimpanzees adhered to the 2002 version of the Guide for the Care and Use of Laboratory Primates issued by KUPRI, which is compatible with the guidelines issued by the National Institutes of Health (Bethesda, MD, USA). The research design was approved by the Animal Welfare and Animal Care Committee of KUPRI and by the Animal Research Committee of Kyoto University. All procedures adhered to the Japanese Act on Welfare and Management of Animals.

### Apparatus

**Figure 1:**
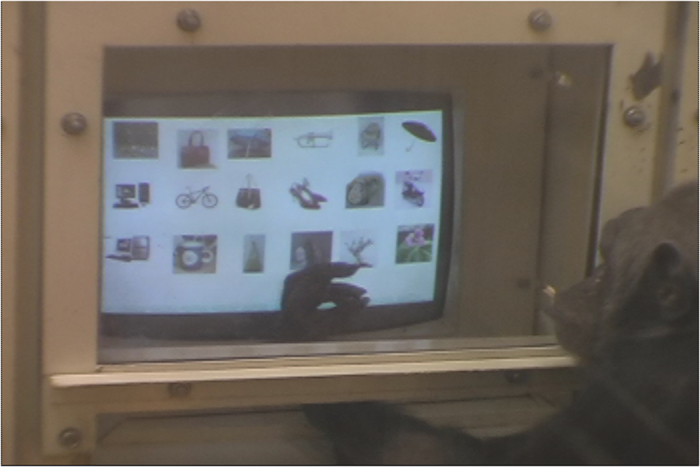
The female chimpanzee Chloe performing the visual search task. She was searching for a chimpanzee face. Photo Courtesy: Masaki Tomonaga (Primate Research Institute, Kyoto University).

Experimental sessions were conducted inside an experimental booth for chimpanzees (1.8 × 2.15 × 1.75 m), which was connected to the outdoor enclosure via overhead walkways. A 21-in color CRT monitor (NEC PC-KH2021) with a capacitive touch-screen device (Microtouch SM-T2) was installed on one side of the booth (Fig. 1). The resolution of the monitor was 640 × 400 pixels. One hundred pixels corresponded to 55 mm. Looking distance was approximately 40 cm. A universal feeder (Biomedica BUF-310) delivered pieces of food (apples or raisins) to a food tray installed below the CRT monitor. All equipment and experimental events were controlled by a PC.

### Procedure

Experiment 1: Efficient searching for chimpanzee faces

### Stimuli

**Figure 2:**
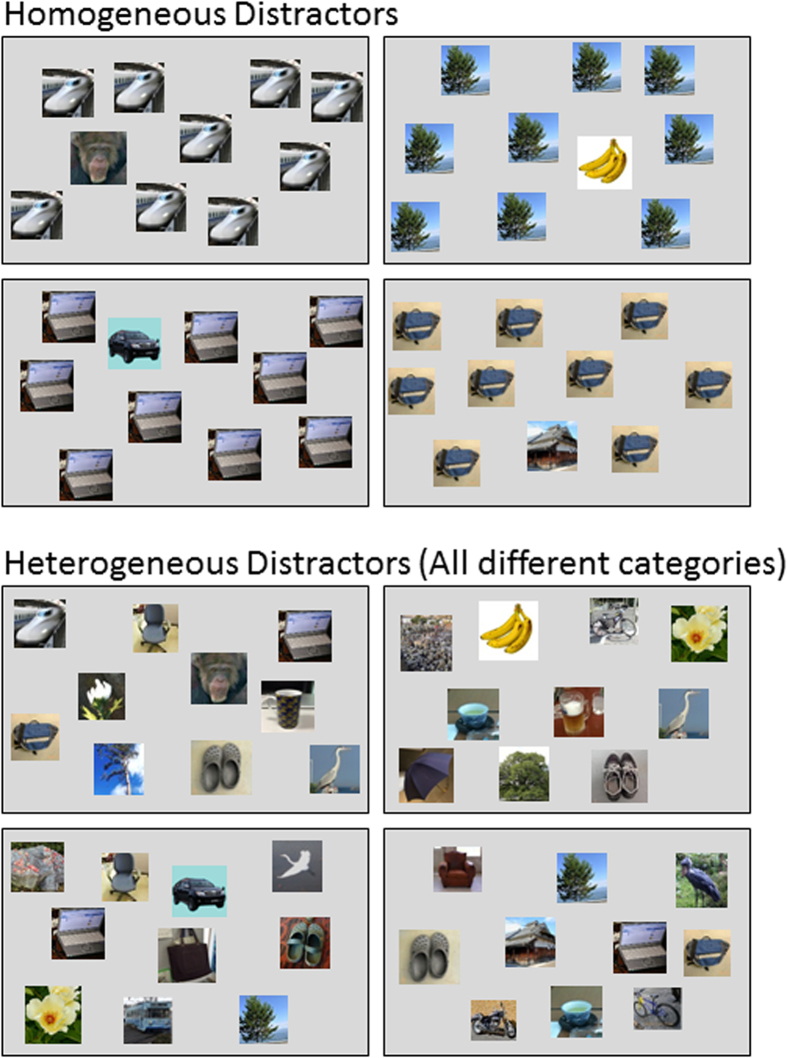
Schematic examples of the search display for each target type. Upper panels: homogeneous distractors used as baseline trials. Lower panels: heterogeneous distractors. Photo Courtesy: Masaki Tomonaga (Primate Research Institute, Kyoto University).

**Figure 3:**
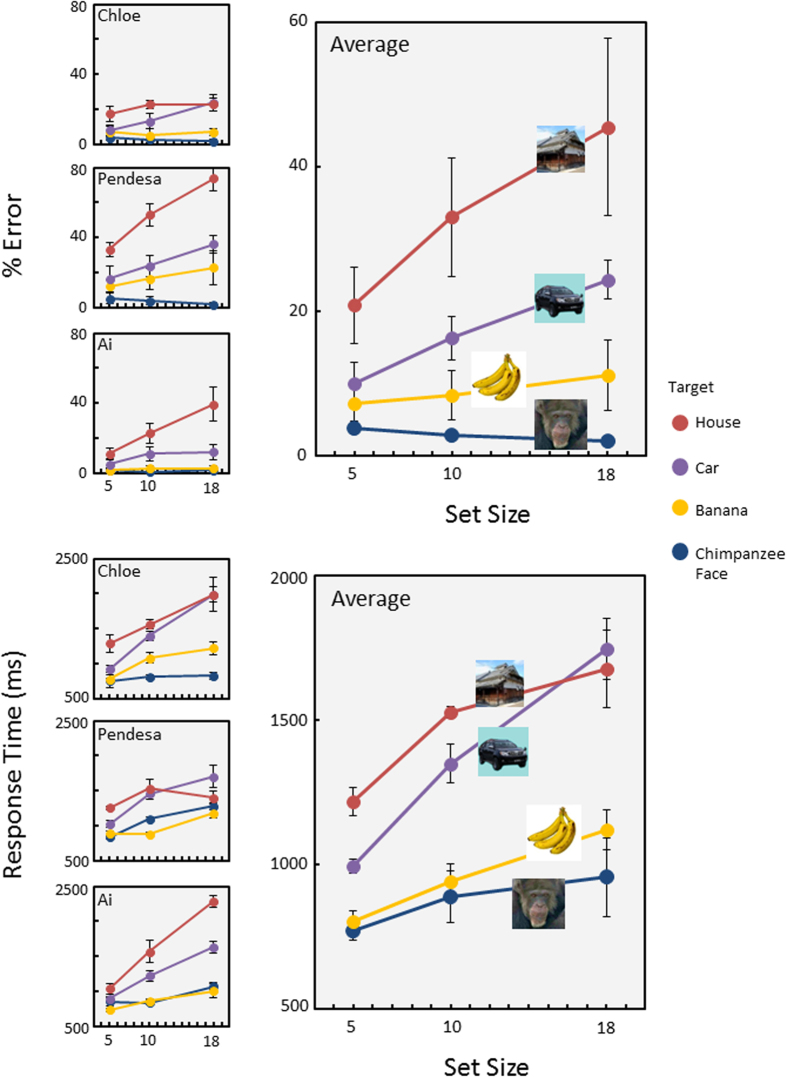
Results of Experiment 1. Upper panels: percentage of errors for each target. Lower panels: response times for correct trials. Error bars show standard errors across chimpanzees. Data from each individual are also shown (error bars show standard errors across sessions). Photo Courtesy: Masaki Tomonaga (Primate Research Institute, Kyoto University).

**Figure 4:**
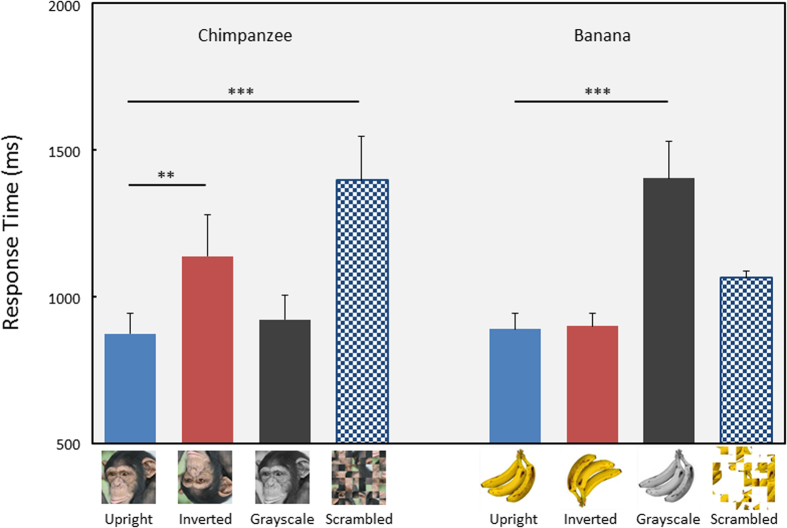
Results of Experiment 2. Response times for correct trials under each condition. Error bars show standard errors across chimpanzees. **p < 0.01, ***p < 0.001. Photo Courtesy: Masaki Tomonaga (Primate Research Institute, Kyoto University).

**Figure 5:**
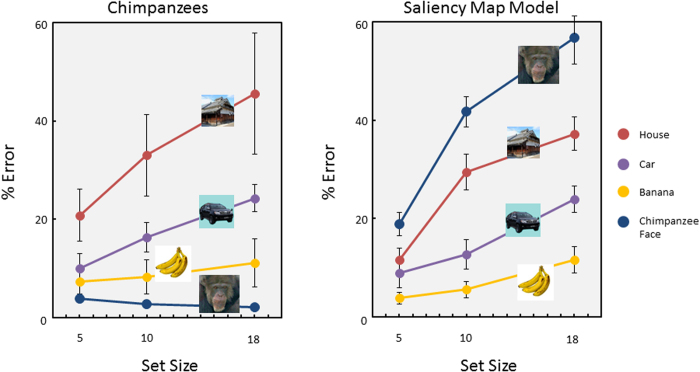
Results of Experiment 3. Left panel: percentage of errors by chimpanzees in Experiment 1. Right panel: Simulation results. Photo Courtesy: Masaki Tomonaga (Primate Research Institute, Kyoto University).

**Figure 6:**
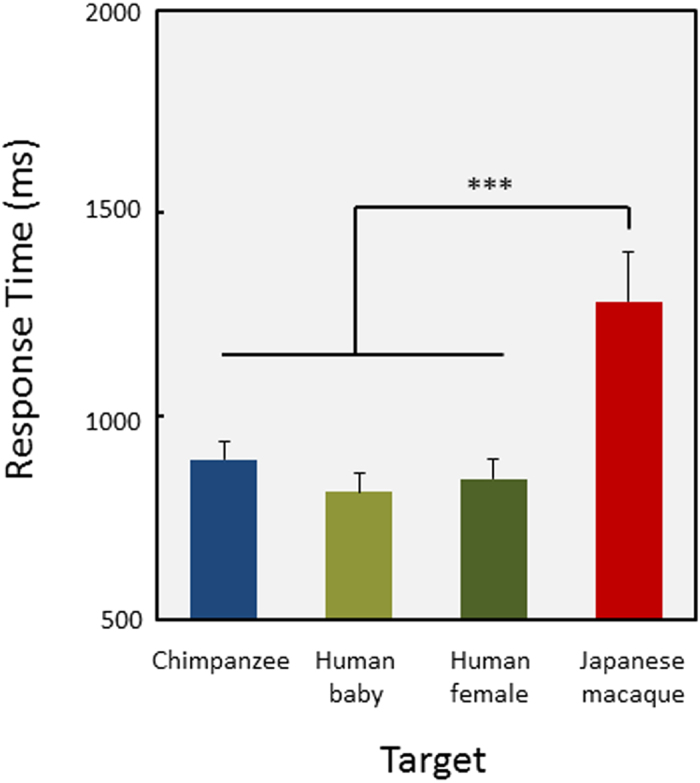
Results of Experiment 4. Response times for correct trials of each target. Error bars show standard errors across chimpanzees. ***p < 0.001.

We used various color pictures (Figs 1 and 2). All pictures were 80 × 80 pixels in size (approximately 44 × 44 mm). We prepared pictures from 22 categories. Among these categories, four were used as target stimuli and the other 18 were used as distractors for the visual search tasks. The target categories were a chimpanzee face, a banana, a car, and a house, respectively. All chimpanzee faces were unfamiliar to the participants. The distractor categories were bag, bicycle, bird, broom, butterfly, chair, cup, fish, flower, guitar, motorbike, PC, rock, shoe, train, tree, trumpet, and umbrella. Eighteen pictures were prepared for each category.

### Visual search task

Chimpanzees were given visual search tasks. Each trial began with the presentation of a 0.5-s beep sound and a blue circle (40 pixels in diameter) at the bottom center of the CRT monitor. When the chimpanzee touched this circle, a search display—which contained a target stimulus and several distractors—was presented (Fig. 1). Each stimulus was presented in a random location. If the chimpanzee touched the target, all stimuli disappeared, a 1-s chime was presented, and a food reward was delivered. If the chimpanzee touched a distractor, all stimuli disappeared, and a 0.5-s buzzer sounded. The intertrial interval was 3 s.

The chimpanzee participants had experienced visual search tasks, as we initially trained them with a simple visual search task. In the initial training, each of the four target categories appeared randomly from trial to trial. In each trial, three identical distractor stimuli (called homogeneous distractors, see also Fig. 2) were presented; thus, the set size ( = the number of stimuli in the search display) was 4. The distractor categories were randomly chosen from trial to trial. Each session consisted of 64 trials, in which each target category appeared 16 times. Each chimpanzee was given this initial training for eight sessions (see Fig. S1 for results). The mean percentages of correct choices for the last two sessions were: 96.9% for Chloe, 90.6% for Pendesa, and 89.8% for Ai, respectively.

After finishing the initial training, a second training was introduced. In this training, we introduced heterogeneous distractors. The distractors differed but were in the same category. Each session consisted of 96 trials, half of which were homogeneous-distractor trials, and the other half were heterogeneous-distractor trials. These two trial types appeared alternately. Unlike in the initial training, the target category was not changed within a session but was changed between sessions. Unlike in the initial training, set size varied at 5, 10, and 18. Each session consisted of 96 trials, and each chimpanzee received eight sessions for each target. The results are shown in Fig. S2. Mean percentages of correct choices for the heterogeneous-distractor trials across the last two sessions were: 91.1% for Chloe, 77.9% for Pendesa, and 93.2% for Ai, respectively.

Immediately after the second training session, all chimpanzees were shifted to the main experiment. In this experiment, heterogeneous distractors were selected from all categories (Fig. 2). As in the second training, the target category was changed between sessions. Each session consisted of 96 trials (48 homogeneous- and 48 heterogeneous-distractor trials). Set size was varied among 5, 10, and 18. Each chimpanzee underwent eight sessions for each target. The first and last sessions were not used for data analyses. Percent errors and response times for correct trials were used for analyses. We used general linear mixed models for data analyses. The fixed effects were Target type and Set size, and the random effects were Participants and Sessions (nested in participants)^32^,^53^,^54^. We used SPSS Advanced Models 14.0J for these analyses (SPSS Inc., Chicago, IL, USA). Graphs for homogeneous-distractor trials are shown in Fig. S3. Raw data are available in Table S1-1. The results of all statistical analyses are provided in Table S2-1.

Experiment 2: Stimulus manipulation test

All three chimpanzees underwent the stimulus manipulation test after finishing Experiment 1. In this test, we prepared four stimulus manipulations, including the “*Upright*” manipulation; i.e., no manipulation (baseline condition). The first treatment was *Inverted*. In this condition, all stimuli in the search display were presented upside down. The second manipulation was *Grayscale*, where all stimuli were changed to grayscale. The third manipulation was *Scrambled*. In this condition, the original stimulus was divided into 8 × 8 matrices (10 pixels each) and randomly scrambled. In this test, we presented only a chimpanzee face and a banana as target stimuli. Each session consisted of 128 trials. Homogeneous- and heterogeneous-distractor trials appeared alternately. All four types of stimulus manipulation were randomly and equally presented within a session. The target category was changed between sessions. Set size was fixed at 10. Each chimpanzee was given eight sessions for each target category, and sessions 2–7 were used for data analyses. General linear mixed models were used to analyze the data. Graphs of each individual are shown in Fig. S4. Raw data and the results of all statistical analyses are available in Tables S1-2 and S2-2.

Experiment 3: Simulation with the saliency map model

We ran a simulation experiment to confirm that the results of Experiment 1 could not be explained by saliency of low-level features. The simulation was based on the “saliency map model”^39^. We used SaliencyToolbox ver.2.2 developed by Walther and Koch^40^. This model localizes salient areas based on low-level features, such as color, brightness, and line orientation, and the most salient area then captures the observer’s attention using a “winner-take-all” principle. The simulation program was run on Matlab R2010. All parameter settings remained unchanged from the initial setting. We prepared 180 heterogeneous-distractor search displays for each target category (4) and set size as input images (3). Thus, the simulation was based on 2,160 trials in total. The simulation was repeated for each search display until the attended location was the target location or until repetition was equal to the set size before attending to the target location. The former case was assigned “correct” detection and the latter case was “error”. These simulation data were also analyzed using a general linear mixed model. Raw data are shown in Table S1-3.

Experiment 4: Visual search for other-species faces

All three chimpanzees participated in this experiment. We prepared three new types of face in addition to the chimpanzee, human baby, human adult female, and Japanese macaque faces. The human face category was familiar to the participating chimpanzees. Furthermore, as there was an enclosure of Japanese macaques next to the chimpanzee enclosure, they had extensive experience visualizing the faces of Japanese macaques. In contrast, the chimpanzees had not seen human babies in their everyday life. We prepared 18 different pictures for each new category. The participants were unfamiliar with these individuals from those categories. Each session consisted of 192 trials. As in previous experiments, homogeneous- and heterogeneous-distractor trials appeared alternately. Set size was again fixed at 10. Unlike in previous experiments, all four target categories appeared randomly and equally. Each chimpanzee underwent six sessions, and the last four sessions were used for data analyses by means of general linear mixed models. Graphs of each individual are shown in Fig. S5. Raw data and the results of all statistical analyses are provided in Tables S1-4 and S2-3.

Experiment 5: Visual search for a profile face and outer features

In the final experiment, we prepared three conditions to investigate the effect of gaze and the roles of inner and outer features of faces. We prepared two conditions. The target categories in the first condition were newly prepared front- and profile-view faces of chimpanzees. The target categories in the second condition were front- and profile-view faces of Japanese adult macaque females.

**Figure 7:**
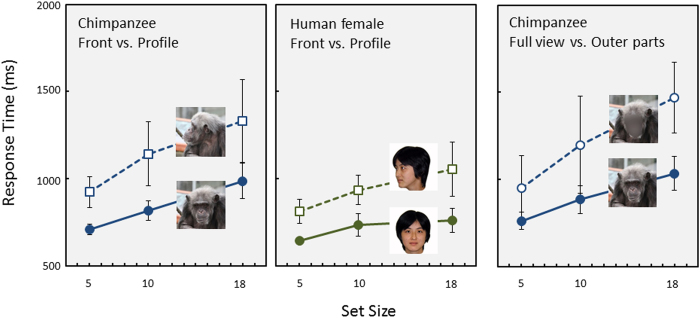
Results of Experiment 5. Response times for correct trials under each condition. Left panel: front- vs. profile-view face of chimpanzee. Center panel: front- vs. profile-view face of human. Right panel: full-view vs. outer parts of chimpanzee face. Error bars show standard errors across chimpanzees. Photo Courtesy: Masaki Tomonaga (Primate Research Institute, Kyoto University).

The target categories were intact upright front-view faces of chimpanzees and those in which the facial area was masked by the average facial area color (Fig. 7). Each session consisted of 96 trials. Unlike in previous experiments, only the heterogeneous-distractor trials were presented. Set size varied among 5, 10, and 18. The two target categories appeared randomly and equally during each session. Each chimpanzee underwent eight sessions under each condition. The first and last sessions of each condition were removed from data analyses using general linear mixed models. Graphs of each individual are shown in Fig. S6. Raw data and the results of all statistical analyses are provided in Tables S1-5 and S2-4.

## Additional Information

**How to cite this article**: Tomonaga, M. and Imura, T. Efficient search for a face by chimpanzees (*Pan troglodytes*). *Sci. Rep.* **5**, 11437; doi: 10.1038/srep11437 (2015).

## Supplementary Material

Supplementary Information
