# Supplementary material for: Efficient search for a face by chimpanzees (Pan troglodytes)
Source: Sci Rep. 2015 Jul 16;5:11437. doi: 10.1038/srep11437 (PMC4504146; doi:10.1038/srep11437)
Supplement: Supplementary Information [file srep11437-s1.pdf]

Supplementary Information

Efficient search for a face by chimpanzees (*Pan troglodytes*)

Masaki TOMONAGA[1,3] and Tomoko IMURA[2]

1) Primate Research Institute, Kyoto University, 41-2 Kanrin, Inuyama, Aichi 484-8506, JAPAN

2) Niigata University of International and Information Studies, 3-1-1 Mizukino, Nishi-ku, Niigata, Niigata 950-2292, JAPAN

3) Corresponding author: tomonaga@pri.kyoto-u.ac.jp

This supplementary information contains 3 sections. In the first section, Tables S1-1 to S1-5, we provide raw data of all 5 experiments. In the second section, Tables S2-1 to S2-4, we provide the results of statistical analyses of all conditions in Experiments 1, 2, 4, and 5. In the third section, we provide supplementary figures (Figures S1 to S6).

Table S1-1(a)
Experiment 1
Efficient Search for the Chimpanzee Face
Participant
Chloe

Target		Distractor Type	Set Size	Session 1	2	3	4	5	6	Average	SEM
Chimpanzee	% correct	Homo	5	100	100	100	100	100	100	100	0
			10	100	93.8	100	100	100	100	99	1
			18	100	100	93.8	100	100	93.8	97.9	1.3
		Hetero	5	100	93.8	93.8	100	93.8	93.8	95.9	1.3
			10	93.8	87.5	100	100	100	100	96.9	2.1
			18	100	100	93.8	93.8	100	100	97.9	1.3
	RT(ms)	Homo	5	553	740	614	619	932	524	664	62
			10	565	614	586	608	694	654	620	19
			18	575	644	570	760	898	588	673	54
		Hetero	5	655	1171	725	745	635	599	755	86
			10	716	774	806	922	941	731	815	39
			18	788	775	1054	793	788	783	830	45
Banana	% correct	Homo	5	100	100	100	100	100	100	100	0
			10	93.8	100	100	100	100	100	99	1
			18	100	100	93.8	100	100	100	99	1
		Hetero	5	93.8	93.8	100	87.5	100	81.3	92.7	3
			10	93.8	75	100	100	100	100	94.8	4.1
			18	93.8	93.8	93.8	100	87.5	87.5	92.7	1.9
	RT(ms)	Homo	5	584	636	582	644	623	542	602	16
			10	653	581	616	748	586	760	657	32
			18	660	618	621	649	594	674	636	12
		Hetero	5	759	836	829	789	761	722	783	18
			10	1286	1040	895	1321	975	991	1085	72
			18	1147	1069	1455	1171	1506	996	1224	85
Car	% correct	Homo	5	100	100	100	100	100	100	100	0
			10	100	100	100	100	100	100	100	0
			18	100	100	100	93.8	93.8	100	97.9	1.3
		Hetero	5	93.8	81.3	87.5	100	87.5	100	91.7	3.1
			10	87.5	68.8	100	93.8	87.5	81.3	86.5	4.4
			18	68.8	68.8	62.5	81.3	81.3	93.8	76.1	4.7
	RT(ms)	Homo	5	730	661	841	747	803	648	738	31
			10	717	627	1103	628	664	617	726	77
			18	609	642	853	643	642	688	680	36
		Hetero	5	879	903	1119	974	874	833	930	42
			10	1179	1235	1527	1491	1624	1342	1400	72
			18	1585	2112	3094	1828	1858	1437	1986	241
House	% correct	Homo	5	100	100	100	100	100	100	100	0
			10	100	100	93.8	100	100	100	99	1
			18	93.8	100	100	93.8	100	93.8	96.9	1.4
		Hetero	5	93.8	81.3	81.3	62.5	87.5	87.5	82.3	4.4
			10	81.3	81.3	68.8	81.3	81.3	68.8	77.1	2.6
			18	87.5	75	75	87.5	75	62.5	77.1	3.8
	RT(ms)	Homo	5	926	614	766	650	728	630	719	48
			10	758	628	681	924	812	817	770	43
			18	640	739	685	689	820	796	728	28
		Hetero	5	933	1827	1147	1351	1139	1343	1290	125
			10	1640	1310	1511	1673	1741	1487	1560	64
			18	1645	2180	1840	2236	1770	2270	1990	110

Note: RT=Response times
SEM=Standard errors of mean

Table S1-1(b)
Experiment 1
Efficient Search for the Chimpanzee Face
Participant
Pendesa

Target		Distractor Type	Set Size	Session 1	2	3	4	5	6	Average	SEM
Chimpanze	% correct	Homo	5	100	100	100	100	100	100	100	0
			10	100	100	100	100	100	100	100	0
			18	100	100	93.8	100	100	100	99	1
		Hetero	5	93.8	81.3	100	100	100	93.8	94.8	3
			10	87.5	100	100	100	93.8	93.8	95.9	2.1
			18	100	100	100	93.8	100	93.8	97.9	1.3
	RT(ms)	Homo	5	675	661	826	735	686	667	708	26
			10	794	862	772	785	778	745	789	16
			18	835	821	856	752	758	725	791	22
		Hetero	5	906	834	829	823	909	758	843	23
			10	1089	1026	1103	1067	1091	1229	1101	28
			18	1225	1357	1385	1160	1401	1172	1283	45
Banana	% correct	Homo	5	100	93.8	93.8	100	100	93.8	96.9	1.4
			10	100	100	100	100	100	100	100	0
			18	100	100	100	100	100	93.8	99	1
		Hetero	5	87.5	93.8	87.5	81.3	75	100	87.5	3.6
			10	87.5	68.8	62.5	93.8	87.5	100	83.4	6
			18	81.3	62.5	37.5	87.5	100	93.8	77.1	9.5
	RT(ms)	Homo	5	637	723	654	641	700	745	683	19
			10	661	645	690	697	669	648	668	9
			18	661	705	740	831	773	678	731	26
		Hetero	5	816	839	819	885	1074	922	893	40
			10	998	816	862	932	840	870	886	27
			18	1153	1196	1497	1004	1224	1041	1186	72
Car	% correct	Homo	5	100	100	100	100	100	100	100	0
			10	100	100	100	100	100	100	100	0
			18	100	100	100	93.8	100	93.8	97.9	1.3
		Hetero	5	50	81.3	87.5	87.5	100	93.8	83.4	7.2
			10	62.5	75	93.8	56.3	75	93.8	76.1	6.3
			18	62.5	43.8	81.3	62.5	62.5	68.8	63.6	5
	RT(ms)	Homo	5	699	660	1059	850	805	758	805	58
			10	856	824	740	992	882	941	873	36
			18	739	848	743	780	785	799	782	16
		Hetero	5	974	956	1083	1228	948	979	1028	45
			10	1269	1515	1342	1606	1725	1291	1458	76
			18	1388	1356	1808	2326	1448	1900	1704	155
House	% correct	Homo	5	93.8	100	100	100	100	100	99	1
			10	100	100	93.8	93.8	100	100	97.9	1.3
			18	93.8	100	100	93.8	100	100	97.9	1.3
		Hetero	5	56.3	68.8	68.8	68.8	81.3	56.3	66.7	3.8
			10	50	31.3	50	25	62.5	62.5	46.9	6.4
			18	25	0	12.5	31.3	43.8	43.8	26.1	7.1
	RT(ms)	Homo	5	820	930	785	914	811	783	841	27
			10	716	786	890	858	856	717	804	31
			18	801	737	837	854	912	838	830	24
		Hetero	5	1246	1307	1182	1258	1192	1373	1260	29
			10	1589	1301	1396	1765	1984	1197	1539	122
			18	1225		1188	1349	1748	1492	1400	93

Note: RT=Response times
SEM=Standard errors of mean

Table S1-1(c)
Experiment 1
Efficient Search for the Chimpanzee Face
Participant
Ai

Target		Distractor Type	Set Size	Session 1	2	3	4	5	6	Average	SEM
Chimpanze	% correct	Homo	5	100	100	100	100	100	100	100	0
			10	100	100	100	100	100	100	100	0
			18	100	100	100	100	100	100	100	0
		Hetero	5	100	93.8	93.8	100	100	100	97.9	1.3
			10	100	93.8	100	100	100	100	99	1
			18	93.8	100	100	100	100	93.8	97.9	1.3
	RT(ms)	Homo	5	675	638	700	735	711	672	689	14
			10	717	845	694	736	776	1002	795	47
			18	798	668	705	760	694	790	736	22
		Hetero	5	765	693	914	743	957	1063	856	59
			10	873	813	784	985	825	798	846	30
			18	891	1166	1190	1085	1121	1025	1080	45
Banana	% correct	Homo	5	100	100	100	100	100	100	100	0
			10	100	100	100	100	100	100	100	0
			18	100	100	100	100	100	100	100	0
		Hetero	5	93.8	100	100	93.8	100	100	97.9	1.3
			10	100	100	93.8	100	93.8	93.8	96.9	1.4
			18	93.8	93.8	100	100	100	93.8	96.9	1.4
	RT(ms)	Homo	5	680	665	683	792	765	783	728	24
			10	714	683	715	853	840	900	784	37
			18	648	743	757	735	828	766	746	24
		Hetero	5	641	762	727	701	837	768	739	27
			10	885	803	794	803	929	967	864	30
			18	913	882	816	868	1108	1474	1010	101
Car	% correct	Homo	5	100	100	100	100	100	100	100	0
			10	100	100	100	100	100	100	100	0
			18	100	100	100	100	100	100	100	0
		Hetero	5	87.5	100	93.8	87.5	100	100	94.8	2.5
			10	87.5	75	81.3	100	100	87.5	88.6	4.1
			18	68.8	93.8	93.8	93.8	93.8	81.3	87.6	4.3
	RT(ms)	Homo	5	799	779	870	737	867	714	794	26
			10	778	805	915	749	1040	726	836	49
			18	682	989	860	739	885	789	824	45
		Hetero	5	861	898	900	871	1025	833	898	27
			10	1234	1025	1351	1105	1528	1138	1230	75
			18	1921	1704	1458	1443	1839	1456	1637	87
House	% correct	Homo	5	100	100	93.8	100	100	100	99	1
			10	100	100	100	100	100	100	100	0
			18	100	100	100	93.8	100	100	99	1
		Hetero	5	81.3	87.5	81.3	100	87.5	93.8	88.6	3
			10	75	56.3	93.8	68.8	87.5	81.3	77.1	5.5
			18	43.8	25	81.3	75	87.5	50	60.4	10
	RT(ms)	Homo	5	1033	866	824	845	770	927	878	38
			10	801	869	1043	931	920	948	919	33
			18	830	1034	813	1046	1050	1133	984	53
		Hetero	5	1182	1305	945	1018	885	907	1040	69
			10	2117	1953	1143	1232	1481	1512	1573	159
			18	2115	2046	2422	2535	2137	2449	2284	85

Note: RT=Response times
SEM=Standard errors of mean

Table S1-2(a)
Experiment 2
Stimulus Manipulation Test
Set Size=10
Participant
Chloe

Target		Distractor Type	Manipulation	Session 1	2	3	4	5	6	Average	SEM
Chimpanze	% correct	Homo	Intact	100	100	100	100	100	100	100	0
			Inverted	100	93.8	93.8	100	100	100	97.9	1.3
			BW	93.8	93.8	100	100	100	100	97.9	1.3
			Scrambled	87.5	100	93.8	100	93.8	100	95.9	2.1
		Hetero	Intact	93.8	100	100	93.8	100	100	97.9	1.3
			Inverted	93.8	100	100	100	100	100	99	1
			BW	100	93.8	93.8	100	93.8	100	96.9	1.4
			Scrambled	6.3	18.8	18.8	25	18.8	12.5	16.7	2.6
	RT(ms)	Homo	Intact	628	547	580	807	715	617	649	39
			Inverted	651	590	1258	713	629	961	800	106
			BW	612	585	737	588	639	582	624	24
			Scrambled	1013	1008	732	863	705	788	852	55
		Hetero	Intact	875	763	723	786	798	730	779	23
			Inverted	1196	929	1043	1022	883	937	1002	46
			BW	790	657	739	739	795	879	767	30
			Scrambled	1985	2562	1020	1457	877	1785	1614	257
Banana	% correct	Homo	Intact	93.8	100	100	87.5	100	93.8	95.9	2.1
			Inverted	100	100	93.8	100	87.5	100	96.9	2.1
			BW	100	100	100	100	100	100	100	0
			Scrambled	93.8	100	100	100	100	100	99	1
		Hetero	Intact	100	87.5	87.5	87.5	100	93.8	92.7	2.5
			Inverted	93.8	81.3	93.8	87.5	100	93.8	91.7	2.6
			BW	56.3	62.5	50	62.5	68.8	68.8	61.5	3
			Scrambled	56.3	43.8	62.5	68.8	62.5	87.5	63.6	5.9
	RT(ms)	Homo	Intact	551	598	641	610	613	641	609	14
			Inverted	702	672	604	584	554	597	619	23
			BW	1063	853	977	999	844	775	919	45
			Scrambled	682	654	598	673	704	814	688	29
		Hetero	Intact	925	764	828	1083	1037	767	901	56
			Inverted	909	1039	999	819	981	1024	962	34
			BW	1619	1289	1175	1332	1154	1370	1323	69
			Scrambled	1218	1333	944	935	978	977	1064	69

Note: RT=Response times
SEM=Standard errors of mean

Table S1-2(b)
Experiment 2
Stimulus Manipulation Test
Set Size=10
Participant
Pendesa

Target		Distractor Type	Manipulation	Session 1	2	3	4	5	6	Average	SEM
Chimpanze	% correct	Homo	Intact	100	100	100	100	100	100	100	0
			Inverted	100	100	100	100	100	100	100	0
			BW	100	100	100	81.3	100	100	96.9	3.1
			Scrambled	87.5	100	100	87.5	93.8	87.5	92.7	2.5
		Hetero	Intact	93.8	100	93.8	100	93.8	100	96.9	1.4
			Inverted	87.5	93.8	81.3	81.3	87.5	93.8	87.5	2.3
			BW	100	93.8	93.8	93.8	100	100	96.9	1.4
			Scrambled	25	43.8	37.5	18.8	25	31.3	30.2	3.8
	RT(ms)	Homo	Intact	732	818	773	696	787	789	766	18
			Inverted	815	926	869	828	924	888	875	19
			BW	833	748	806	713	812	887	800	25
			Scrambled	1021	980	913	897	1392	1075	1046	74
		Hetero	Intact	932	935	924	1173	991	1303	1043	65
			Inverted	1400	1734	1324	1646	1430	1328	1477	70
			BW	1119	1017	932	1363	1296	956	1114	74
			Scrambled	1108	1646	1932	1987	1353	1218	1541	152
Banana	% correct	Homo	Intact	87.5	100	100	100	100	100	97.9	2.1
			Inverted	100	100	93.8	100	100	93.8	97.9	1.3
			BW	75	93.8	93.8	93.8	93.8	100	91.7	3.5
			Scrambled	100	100	100	100	100	87.5	97.9	2.1
		Hetero	Intact	100	81.3	75	75	100	93.8	87.5	4.8
			Inverted	81.3	81.3	81.3	68.8	93.8	75	80.3	3.4
			BW	18.8	18.8	37.5	50	56.3	56.3	39.6	7.2
			Scrambled	81.3	62.5	81.3	93.8	93.8	81.3	82.3	4.7
	RT(ms)	Homo	Intact	752	723	771	679	983	715	771	44
			Inverted	711	760	680	772	722	765	735	15
			BW	1082	1323	1113	1341	1230	1135	1204	45
			Scrambled	817	802	676	722	760	732	752	21
		Hetero	Intact	837	994	1094	1227	972	857	997	60
			Inverted	980	1046	735	988	844	1057	942	52
			BW	2567	1632	1582	1204	1553	1657	1699	186
			Scrambled	996	872	1130	1015	1025	1016	1009	34

Note: RT=Response times
SEM=Standard errors of mean

Table S1-2(b)
Experiment 2
Stimulus Manipulation Test
Set Size=10
Participant
Pendesa

Target		Distractor Type	Manipulation	Session 1	2	3	4	5	6	Average	SEM
Chimpanze	% correct	Homo	Intact	100	100	100	100	100	100	100	0
			Inverted	100	100	100	100	100	100	100	0
			BW	100	100	100	81.3	100	100	96.9	3.1
			Scrambled	87.5	100	100	87.5	93.8	87.5	92.7	2.5
		Hetero	Intact	93.8	100	93.8	100	93.8	100	96.9	1.4
			Inverted	87.5	93.8	81.3	81.3	87.5	93.8	87.5	2.3
			BW	100	93.8	93.8	93.8	100	100	96.9	1.4
			Scrambled	25	43.8	37.5	18.8	25	31.3	30.2	3.8
	RT(ms)	Homo	Intact	732	818	773	696	787	789	766	18
			Inverted	815	926	869	828	924	888	875	19
			BW	833	748	806	713	812	887	800	25
			Scrambled	1021	980	913	897	1392	1075	1046	74
		Hetero	Intact	932	935	924	1173	991	1303	1043	65
			Inverted	1400	1734	1324	1646	1430	1328	1477	70
			BW	1119	1017	932	1363	1296	956	1114	74
			Scrambled	1108	1646	1932	1987	1353	1218	1541	152
Banana	% correct	Homo	Intact	87.5	100	100	100	100	100	97.9	2.1
			Inverted	100	100	93.8	100	100	93.8	97.9	1.3
			BW	75	93.8	93.8	93.8	93.8	100	91.7	3.5
			Scrambled	100	100	100	100	100	87.5	97.9	2.1
		Hetero	Intact	100	81.3	75	75	100	93.8	87.5	4.8
			Inverted	81.3	81.3	81.3	68.8	93.8	75	80.3	3.4
			BW	18.8	18.8	37.5	50	56.3	56.3	39.6	7.2
			Scrambled	81.3	62.5	81.3	93.8	93.8	81.3	82.3	4.7
	RT(ms)	Homo	Intact	752	723	771	679	983	715	771	44
			Inverted	711	760	680	772	722	765	735	15
			BW	1082	1323	1113	1341	1230	1135	1204	45
			Scrambled	817	802	676	722	760	732	752	21
		Hetero	Intact	837	994	1094	1227	972	857	997	60
			Inverted	980	1046	735	988	844	1057	942	52
			BW	2567	1632	1582	1204	1553	1657	1699	186
			Scrambled	996	872	1130	1015	1025	1016	1009	34

Note: RT=Response times
SEM=Standard errors of mean

Table S1-3
Experiment 3
Simulation with Saliency Map Model

Distractor			Repetition (correct position)																			
Type	TGT	Set Size	1	2	3	4	5	6	7	8	9	10	11	12	13	14	15	16	17	18	Average	SEM
Hetero	Chimpanze	5	0	10	20	40	20	10	30	30	30	10	30	10	20	10	20	20	10	20	18.9	2.4
		10	50	40	60	50	60	30	20	50	50	50	40	40	40	50	40	40	30	10	41.7	3.1
		18	60	50	50	70	50	50	70	80	80	80	100	70	10	50	40	20	50	40	56.7	5.2
	Banana	5	10	0	0	0	10	0	10	10	0	10	0	0	0	0	10	10	0	0	3.9	1.2
		10	0	10	0	0	20	10	10	10	0	0	0	0	0	20	0	10	0	10	5.6	1.7
		18	10	0	30	20	0	0	30	20	0	10	10	0	20	20	0	30	10	0	11.7	2.7
	Car	5	40	10	0	10	10	10	30	0	0	30	0	0	0	0	10	0	0	10	8.9	2.9
		10	30	20	30	0	10	10	10	10	20	30	40	0	0	0	10	10	0	0	12.8	3
		18	10	30	40	30	20	20	20	40	40	20	30	20	10	40	0	20	20	20	23.9	2.7
	House	5	10	0	20	20	10	0	40	10	10	10	10	20	0	0	20	10	10	10	11.7	2.3
		10	60	30	20	30	10	10	30	40	30	40	60	20	20	20	50	30	10	20	29.4	3.7
		18	20	60	20	50	30	30	60	40	50	40	50	60	30	20	30	30	20	30	37.2	3.4

Note: Data for each cell are based on 10 simulation trials

Table S1-4(a)
Experiment 4
Visual Search for the Other-Species Face
Set Size=10
Participant
Chloe

	Distractor Type	Target	Session 1	2	3	4	Average	SEM
% correct	Homo	Chimpanzee	96.3	100	100	100	99.1	0.9
		Human Baby	100	100	100	100	100	0
		Human Female	100	100	100	100	100	0
		Japanese Macaque	100	100	100	100	100	0
	Hetero	Chimpanzee	98	96	95.8	98	97	0.6
		Human Baby	100	100	100	100	100	0
		Human Female	100	100	95	100	98.8	1.3
		Japanese Macaque	78.3	92.9	95	78.6	86.2	4.5
RT(ms)	Homo	Chimpanzee	716	574	577	641	627	33
		Human Baby	603	538	610	535	572	20
		Human Female	615	620	652	645	633	9
		Japanese Macaque	787	687	728	628	708	34
	Hetero	Chimpanzee	971	750	735	789	811	54
		Human Baby	756	778	707	811	763	22
		Human Female	741	611	768	787	727	40
		Japanese Macaque	1120	1272	1368	1234	1249	51

Note: RT=Response times
SEM=Standard errors of mean

Table S1-4(b)
Experiment 4
Visual Search for the Other-Species Face
Set Size=10
Participant
Pendesa

	Distractor Type	Target	Session 1	2	3	4	Average	SEM
% correct	Homo	Chimpanzee	100	98.1	100	100	99.5	0.5
		Human Baby	100	100	100	100	100	0
		Human Female	100	100	100	100	100	0
		Japanese Macaque	92.9	92.9	94.4	100	95.1	1.7
	Hetero	Chimpanzee	100	100	95.8	95.7	97.9	1.2
		Human Baby	87.3	100	92.9	100	95.1	3.1
		Human Female	100	100	100	85	96.3	3.8
		Japanese Macaque	86.7	92.9	95	100	93.7	2.8
RT(ms)	Homo	Chimpanzee	694	725	806	729	739	24
		Human Baby	667	676	762	682	697	22
		Human Female	672	911	624	700	727	63
		Japanese Macaque	839	912	937	1002	923	34
	Hetero	Chimpanzee	1045	996	971	987	1000	16
		Human Baby	945	1012	863	889	927	33
		Human Female	893	993	851	936	918	30
		Japanese Macaque	1642	1987	1357	1253	1560	164

Note: RT=Response times
SEM=Standard errors of mean

Table S1-4(c)
Experiment 4
Visual Search for the Other-Species Face
Set Size=10
Participant
Ai

	Distractor Type	Target	Session 1	2	3	4	Average	SEM
% correct	Homo	Chimpanzee	93.2	95.8	95.3	97.9	95.6	1
		Human Baby	100	100	95.2	100	98.8	1.2
		Human Female	100	100	100	100	100	0
		Japanese Macaque	100	100	100	100	100	0
	Hetero	Chimpanzee	100	100	100	100	100	0
		Human Baby	89.5	94	100	96	94.9	2.2
		Human Female	88.9	100	94.4	100	95.8	2.7
		Japanese Macaque	86.7	95	95	100	94.2	2.8
RT(ms)	Homo	Chimpanzee	826	817	808	792	811	7
		Human Baby	659	778	777	801	754	32
		Human Female	855	672	728	625	720	50
		Japanese Macaque	697	717	756	629	700	27
	Hetero	Chimpanzee	713	719	834	740	752	28
		Human Baby	964	908	842	759	868	44
		Human Female	712	809	723	771	754	22
		Japanese Macaque	902	886	922	865	894	12

Note: RT=Response times
SEM=Standard errors of mean

Table S1-5(a)
Experiment 5
Visual Search for Profile Face and Outer Features
Distractor Type: Heterogeneous
Participant
Chloe

			Session							
	Target	Set Size	1	2	3	4	5	6	Average	SEM
% correct	Chimpanzee	5	100	100	93.8	100	100	87.5	96.9	2.1
	Front	10	93.8	100	93.8	100	100	87.5	95.9	2.1
		18	100	100	100	100	87.5	100	97.9	2.1
	Chimpanzee	5	100	87.5	100	100	100	100	97.9	2.1
	Profile	10	93.8	93.8	81.3	87.5	93.8	87.5	89.6	2.1
		18	87.5	87.5	87.5	87.5	87.5	81.3	86.5	1
RT(ms)	Chimpanzee	5	707	681	629	635	669	620	657	14
	Front	10	730	714	848	724	658	842	753	31
		18	931	876	828	859	757	734	831	30
	Chimpanzee	5	918	808	861	770	797	772	821	24
	Profile	10	897	1121	907	1103	878	843	958	50
		18	1150	1231	1069	1109	1172	1214	1158	25
% correct	Japanese female	5	100	93.8	93.8	93.8	100	100	96.9	1.4
	Front	10	100	100	93.8	100	100	100	99	1
		18	100	93.8	100	100	100	93.8	97.9	1.3
	Japanese female	5	100	93.8	100	100	93.8	100	97.9	1.3
	Profile	10	100	93.8	87.5	100	100	100	96.9	2.1
		18	100	100	87.5	93.8	100	100	96.9	2.1
RT(ms)	Japanese female	5	679	597	563	571	635	640	614	18
	Front	10	705	670	603	620	577	558	622	23
		18	755	626	601	725	643	607	660	26
	Japanese female	5	697	666	673	748	635	708	688	16
	Profile	10	838	799	885	758	725	814	803	23
		18	895	863	791	891	760	1064	877	44
% correct	Chimpanzee	5	100	100	93.8	93.8	100	93.8	96.9	1.4
	Intact	10	93.8	93.8	100	100	93.8	100	96.9	1.4
		18	87.5	100	100	100	87.5	100	95.8	2.6
	Chimpanzee	5	100	100	87.5	87.5	100	93.8	94.8	2.5
	Outer parts	10	100	100	100	75	93.8	87.5	92.7	4.1
		18	87.5	93.8	93.8	100	75	100	91.7	3.8
RT(ms)	Chimpanzee	5	738	634	605	631	724	774	684	28
	Intact	10	795	694	719	795	738	721	744	17
		18	902	1025	778	693	877	1005	880	52
	Chimpanzee	5	835	684	689	736	646	869	743	37
	Outer parts	10	1131	896	883	808	833	916	911	47
		18	1013	1266	1217	938	1094	1559	1181	91

Note: RT=Response times
SEM=Standard errors of mean

Table S1-5(b)
Experiment 5
Visual Search for Profile Face and Outer Features
Distractor Type: Heterogeneous
Participant
Pendesa

	Target	Set Size	Session 1	2	3	4	5	6	Average	SEM
% correct	Chimpanzee	5	100	100	100	100	100	100	100	0
	Front	10	100	100	100	100	100	93.8	99	1
		18	93.8	93.8	87.5	93.8	100	93.8	93.8	1.6
	Chimpanzee	5	100	87.5	100	93.8	100	87.5	94.8	2.5
	Profile	10	75	81.3	87.5	100	100	75	86.5	4.7
		18	81.3	75	93.8	81.3	93.8	93.8	86.5	3.4
RT(ms)	Chimpanzee	5	831	791	766	717	708	740	759	19
	Front	10	808	886	928	1087	816	1038	927	47
		18	1160	1196	1343	1120	1103	1134	1176	36
	Chimpanzee	5	1174	917	1378	969	959	1196	1099	74
	Profile	10	1519	1507	1465	1212	1624	1730	1510	71
		18	1903	1506	1811	2015	2055	1510	1800	99
% correct	Japanese female	5	93.8	100	100	100	100	100	99	1
	Front	10	100	100	100	100	100	100	100	0
		18	100	93.8	100	93.8	100	100	97.9	1.3
	Japanese female	5	87.5	93.8	100	100	93.8	100	95.9	2.1
	Profile	10	93.8	81.3	93.8	100	100	93.8	93.8	2.8
		18	81.3	100	93.8	93.8	87.5	93.8	91.7	2.6
RT(ms)	Japanese female	5	671	673	658	632	650	705	665	10
	Front	10	904	946	800	784	862	814	852	26
		18	846	881	902	902	785	1012	888	31
	Japanese female	5	905	962	870	884	1005	913	923	21
	Profile	10	1250	1299	1071	1008	925	979	1089	62
		18	1396	1433	1763	1115	1322	1155	1364	95
% correct	Chimpanzee	5	100	100	100	100	100	100	100	0
	Intact	10	93.8	87.5	100	93.8	100	81.3	92.7	3
		18	87.5	93.8	93.8	93.8	100	93.8	93.8	1.6
	Chimpanzee	5	75	75	93.8	68.8	81.3	93.8	81.3	4.3
	Outer parts	10	81.3	75	75	81.3	75	93.8	80.2	3
		18	75	31.3	68.8	75	62.5	68.8	63.6	6.7
RT(ms)	Chimpanzee	5	1012	817	848	876	809	755	853	36
	Intact	10	1080	1126	1107	933	970	907	1021	39
		18	1295	1191	1231	1259	1208	1113	1216	26
	Chimpanzee	5	1887	1470	1036	1408	1205	907	1319	143
	Outer parts	10	1828	1809	1720	1970	1746	1455	1755	70
		18	1789	2103	2042	2202	1474	1564	1862	123

Note: RT=Response times
SEM=Standard errors of mean

Table S1-5(c)
Experiment 5
Visual Search for Profile Face and Outer Features
Distractor Type: Heterogeneous
Participant
Ai

			Session							
	Target	Set Size	1	2	3	4	5	6	Average	SEM
% correct	Chimpanzee	5	100	100	100	100	100	100	100	0
	Front	10	100	93.8	100	100	100	100	99	1
		18	100	100	100	100	93.8	100	99	1
	Chimpanzee	5	87.5	100	93.8	100	100	100	96.9	2.1
	Profile	10	93.8	93.8	93.8	93.8	100	87.5	93.8	1.6
		18	100	100	100	93.8	81.3	100	95.9	3.1
RT(ms)	Chimpanzee	5	681	805	646	676	772	697	713	25
	Front	10	837	830	752	755	727	723	771	21
		18	932	1028	902	935	1033	887	953	26
	Chimpanzee	5	712	1162	778	730	755	976	852	73
	Profile	10	939	1026	1043	972	906	876	960	27
		18	1193	979	914	1122	964	1016	1031	43
% correct	Japanese female	5	100	100	100	100	100	100	100	0
	Front	10	100	100	100	100	100	100	100	0
		18	93.8	100	93.8	100	100	100	97.9	1.3
	Japanese female	5	100	100	100	100	100	100	100	0
	Profile	10	100	100	100	93.8	100	100	99	1
		18	93.8	93.8	87.5	93.8	81.3	93.8	90.7	2.1
RT(ms)	Japanese female	5	598	609	649	685	644	718	651	19
	Front	10	687	675	680	719	677	936	729	42
		18	697	727	798	742	761	683	735	17
	Japanese female	5	779	724	831	773	805	1072	831	50
	Profile	10	796	877	1126	928	893	859	913	46
		18	759	858	870	1084	1062	907	923	51
% correct	Chimpanzee	5	93.8	100	93.8	100	100	93.8	96.9	1.4
	Intact	10	93.8	93.8	87.5	93.8	100	100	94.8	1.9
		18	93.8	93.8	87.5	100	93.8	100	94.8	1.9
	Chimpanzee	5	81.3	93.8	93.8	87.5	100	100	92.7	3
	Outer parts	10	62.5	93.8	87.5	100	93.8	93.8	88.6	5.5
		18	81.3	93.8	93.8	87.5	87.5	81.3	87.5	2.3
RT(ms)	Chimpanzee	5	895	779	735	711	698	682	750	32
	Intact	10	1063	906	877	857	880	771	892	39
		18	994	840	981	1328	964	925	1005	68
	Chimpanzee	5	925	771	756	826	724	755	793	30
	Outer parts	10	1211	994	836	907	862	758	928	65
		18	1515	1421	1140	1298	1391	1458	1371	55

Note: RT=Response times
SEM=Standard errors of mean

Table S2-1. Results of General Mixed Model Analysis for Experiment 1

% Error

Homogeneous distractors

Factor	df1	df2	F	p
Target type	3	202	2.13	0.098
Set size	2	202	4.87	0.009
Interaction	6	202	0.91	0.489

Heterogeneous distractors

Factor	df1	df2	F	p
Target type	***3***	***187***	***68.94***	***4.550E-30***
Set size	2	187	14.12	1.947E-06
Interaction	6	187	4.57	2.362E-04

Multiple Comparisons for Target type

	ChimpFace	Banana	Car
Banana	0.045		
Car	0	0.003	
House	0	0	0

Response Times

Homogeneous distractors

Factor	df1	df2	F	p
Target type	***3***	***187***	***26.78***	***1.858E-14***
Set size	2	187	2.65	0.074
Interaction	6	187	0.67	0.671

Multiple Comparisons for Target type

	ChimpFace	Banana	Car
Banana	0.594		
Car	0.001	0	
House	0	0	0.045

Heterogeneous distractors

Factor	df1	df2	F	p
Target type	***3***	***204***	***83.42***	***2.936E-35***
Set size	2	204	83.79	2.733E-27
Interaction	6	204	5.92	1.021E-05

Multiple Comparisons for Target type

	ChimpFace	Banana	Car
Banana	0.991		
Car	0	0	
House	0	0	0.001

Table S2-2. Results of General Mixed Model Analysis for Experiment 2

% Error

Homogeneous distractors

Factor	df1	df2	F	p
Target type	1	136	0.679	0.411
Manipulation	3	136	6.369	4.518E-04
Interaction	***3***	***136***	***6.59***	***3.426E-04***

Multiple Comparisons for Target type X Manipulation

		Upright	Inverted	Grayscale
Chimpanzee	Inverted	0.998		
	Grayscale	0.649	0.923	
	Scrambled	0	0	0.001

		Upright	Inverted	Grayscale
Banana	Inverted	1.000		
	Grayscale	0.809	0.649	
	Scrambled	1.000	1.000	0.809

Heterogeneous distractors

Factor	df1	df2	F	p
Target type	1	158	0.38	0.537
Manipulation	3	158	131.02	1.175E-42
Interaction	***3***	***158***	***52.73***	***1.131E-23***

Multiple Comparisons for Target type X Manipulation

		Upright	Inverted	Grayscale
Chimpanzee	Inverted	0.892		
	Grayscale	1.000	0.977	
	Scrambled	0	0	0

		Upright	Inverted	Grayscale
Banana	Inverted	0.987		
	Grayscale	0	0	
	Scrambled	0	0	1.000

Table S2-2. Results of General Mixed Model Analysis for Experiment 2 (continued)

Response Times

Homogeneous distractors

Factor	df1	df2	F	p
Target type	1	134	0	1.000
Manipulation	3	134	18.68	3.503E-10
Interaction	***3***	***134***	***27.83***	***4.702E-14***

Multiple Comparisons for Target type X Manipulation

		Upright	Inverted	Grayscale
Chimpanzee	Inverted	0.132		
	Grayscale	1.000	0.283	
	Scrambled	0	0.011	0

		Upright	Inverted	Grayscale
Banana	Inverted	1.000		
	Grayscale	0	0	
	Scrambled	0.136	0.206	0

Heterogeneous distractors

Factor	df1	df2	F	p
Target type	1	140	0.57	0.450
Manipulation	3	140	19.01	2.126E-10
Interaction	***3***	***140***	***25.6***	***2.907E-13***

Multiple Comparisons for Target type X Manipulation

		Upright	Inverted	Grayscale
Chimpanzee	Inverted	0.002		
	Grayscale	0.99	0.015	
	Scrambled	0	0	0

		Upright	Inverted	Grayscale
Banana	Inverted	0.993		
	Grayscale	0	0	
	Scrambled	0.118	0.393	0

Table S2-3. Results of General Mixed Model Analysis for Experiment 4

% Error

Homogeneous distractors

Factor	df1	df2	F	p
Target type	***3***	***42***	***3.14***	***0.035***

Multiple Comparisons for Target type

	Chimp	Baby	Human
Baby	0.974		
Human	0.974	1.000	
Monkey	0.329	0.066	0.066

Heterogeneous distractors

Factor	df1	df2	F	p
Target type	***3***	***33***	***6.20***	***0.002***

Multiple Comparisons for Target type

	Chimp	Baby	Human
Baby	1.000		
Human	0.981	0.941	
Monkey	0.006	0.004	0.039

Response Times

Homogeneous distractors

Factor	df1	df2	F	p
Target type	***3***	***42***	***6.43***	***0.001***

Multiple Comparisons for Target type

	Chimp	Baby	Human
Baby	0.92		
Human	0.998	0.997	
Monkey	0.025	0.002	0.007

Heterogeneous distractors

Factor	df1	df2	F	p
Target type	***3***	***33***	***35.70***	***1.804E-10***

Multiple Comparisons for Target type

	Chimp	Baby	Human
Baby	0.499		
Human	0.752	1.000	
Monkey	0	0	0

Table S2-4. Results of General Mixed Model Analysis for Experiment 5

Chimpanzee Face: Front view vs. profile

% Error

Factor	df1	df2	F	p
Target type	1	100	30.92	2.248E-07
Set size	2	100	10.05	1.053E-04
Interaction	2	100	2.42	0.094

Response Times

Factor	df1	df2	F	p
Target type	1	118	107.02	3.039E-18
Set size	2	118	48.04	5.467E-16
Interaction	2	118	1.69	0.189

Chimpanzee Face: Full face vs. outer parts

% Error

Factor	df1	df2	F	p
Target type	1	95	33.47	9.241E-08
Set size	2	95	5.1	7.880E-03
Interaction	2	95	1.55	0.217

Response Times

Factor	df1	df2	F	p
Target type	1	95	86.27	5.489E-15
Set size	2	95	48.02	3.875E-15
Interaction	2	95	4.72	0.011

Human Face: Front view vs. profile

% Error

Factor	df1	df2	F	p
Target type	1	95	23.6	4.658E-06
Set size	2	95	6.65	1.975E-03
Interaction	2	95	3.19	0.046

Response Times

Factor	df1	df2	F	p
Target type	1	95	114.16	5.744E-18
Set size	2	95	27.42	3.990E-10
Interaction	2	95	3.94	0.023

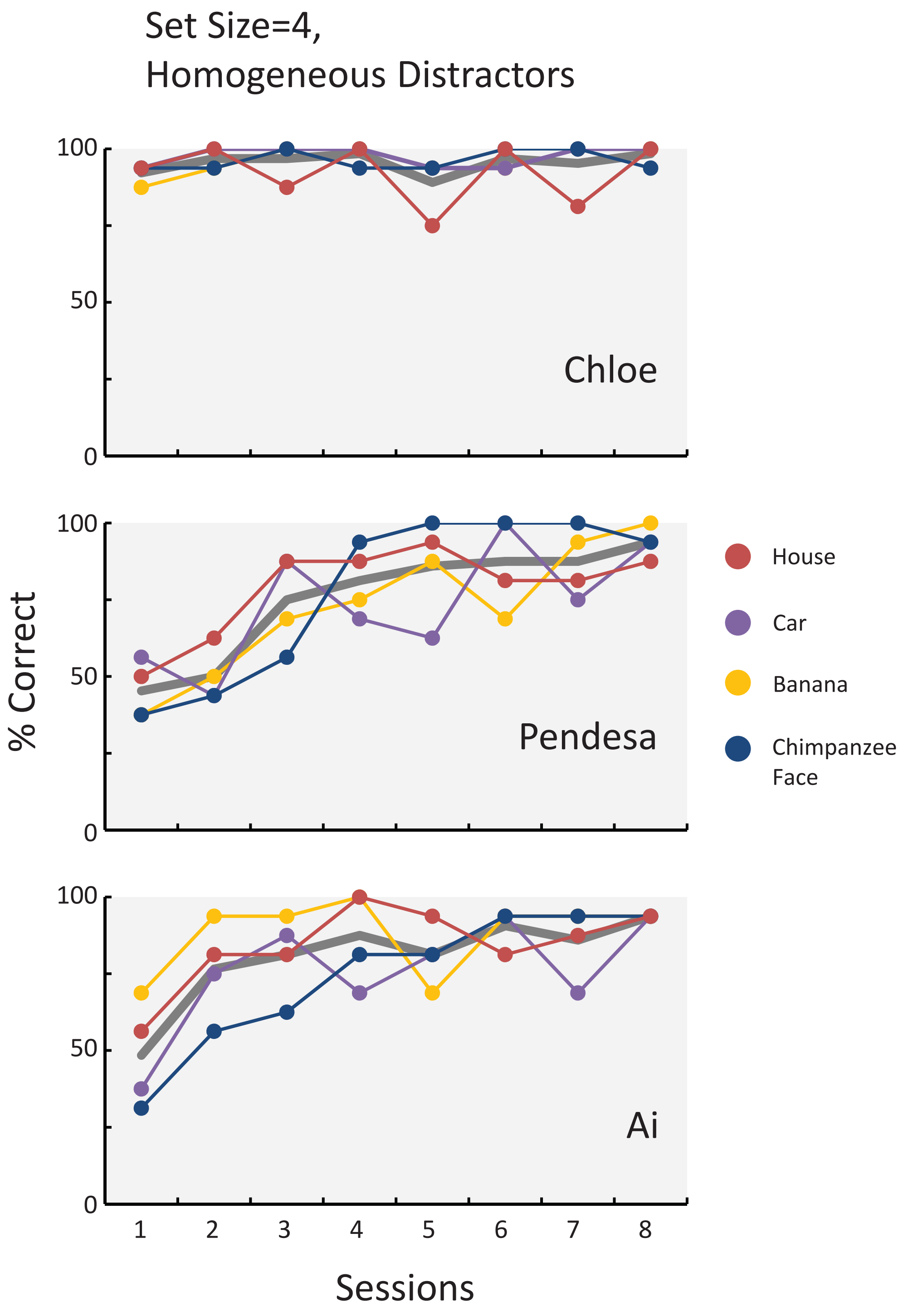

Figure S1. The results of the initial training of Experiment 1. Percentage of correct trials for each target category across sessions are shown.

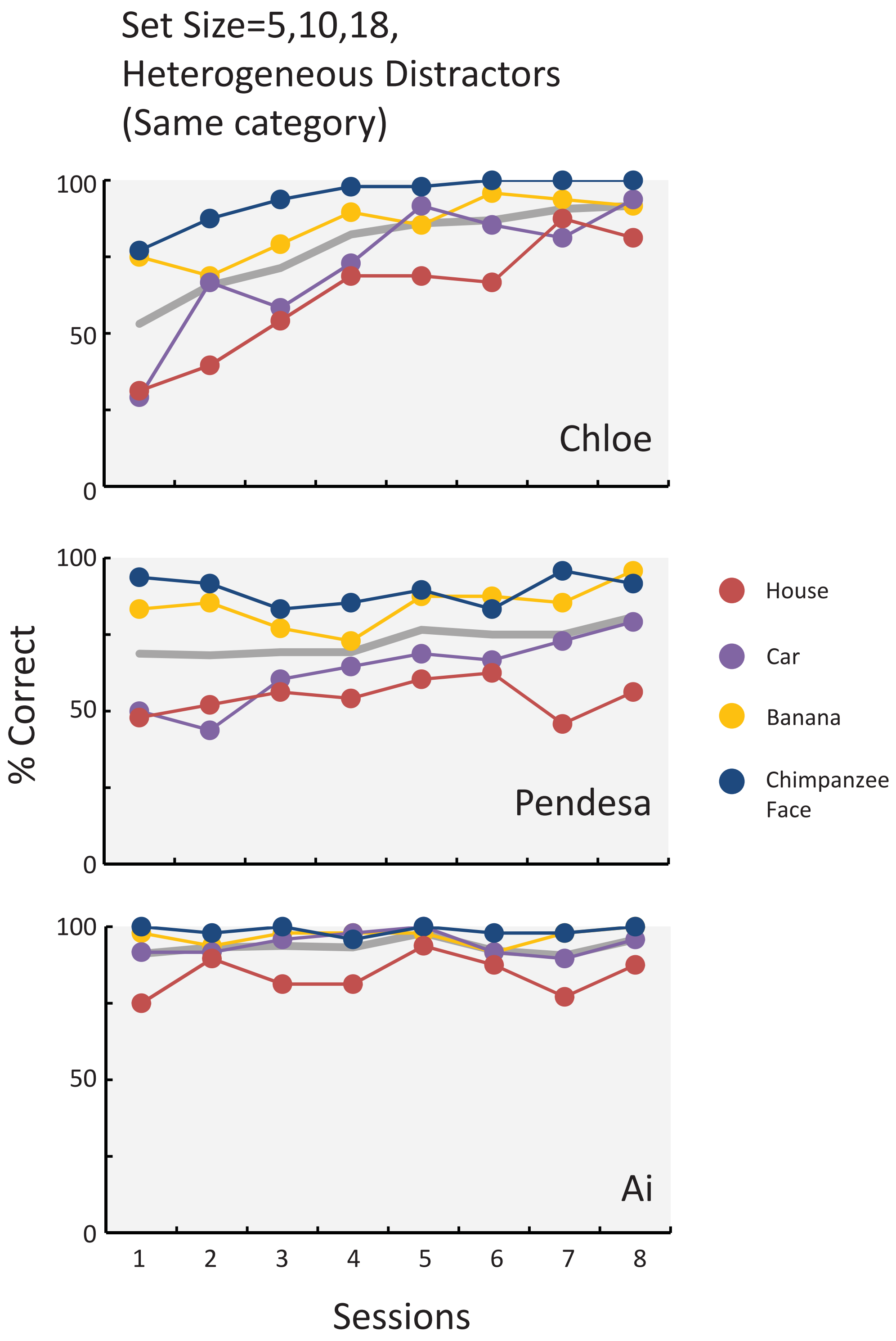

Figure S2. The results of the second training of Experiment 1. Percentage of correct trials for each target category (averaged across set sizes) across sessions are shown.

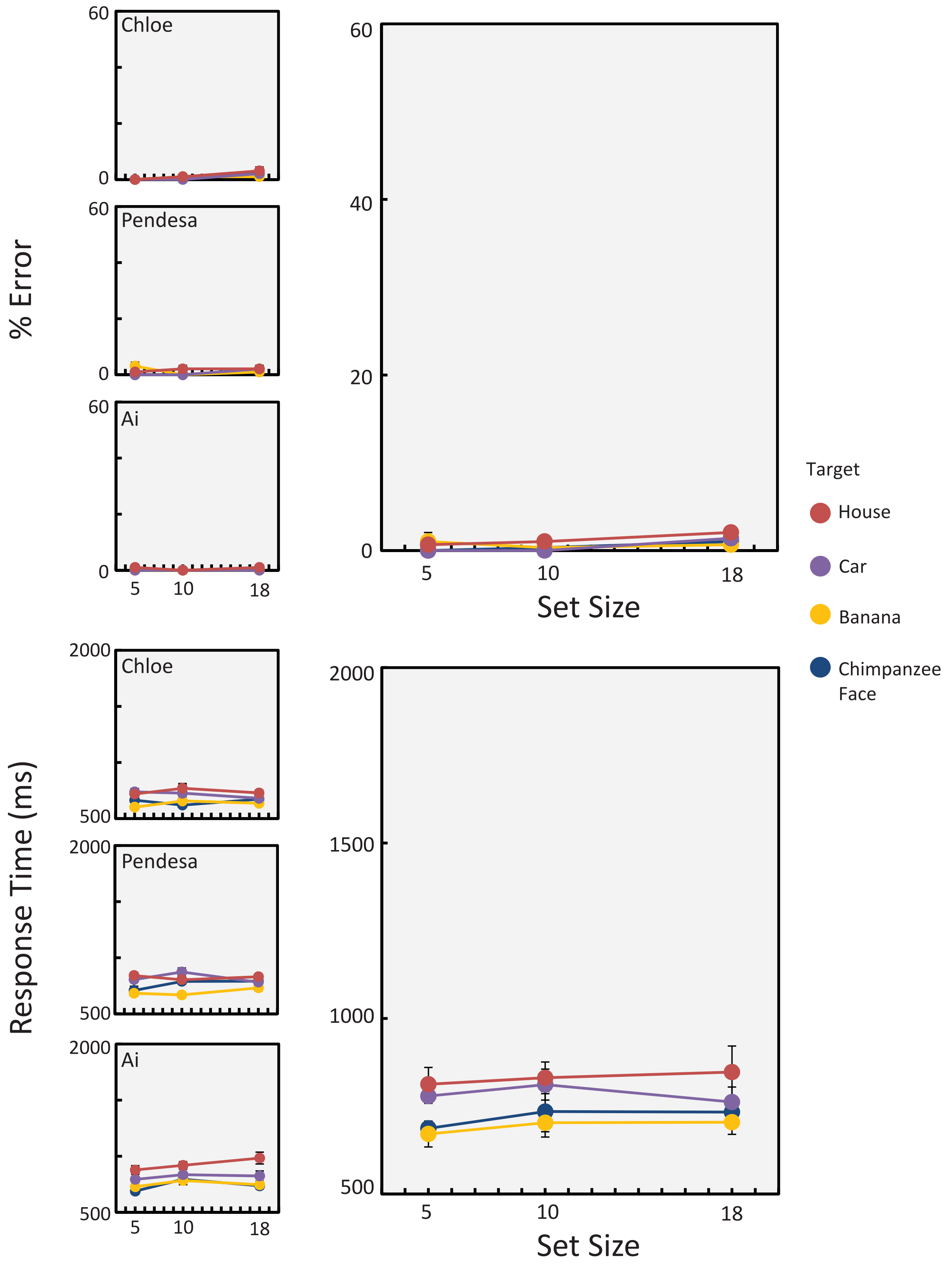

Figure S3. Results of Experiment 1 (Homogeneous-distractor trials). Upper panels: percentage of error trials for each target. Lower panels: response times on correct trials. Error bars show standard errors of mean across chimpanzees. Data from each individual are also shown (Error bars show standard errors of mean across sessions).

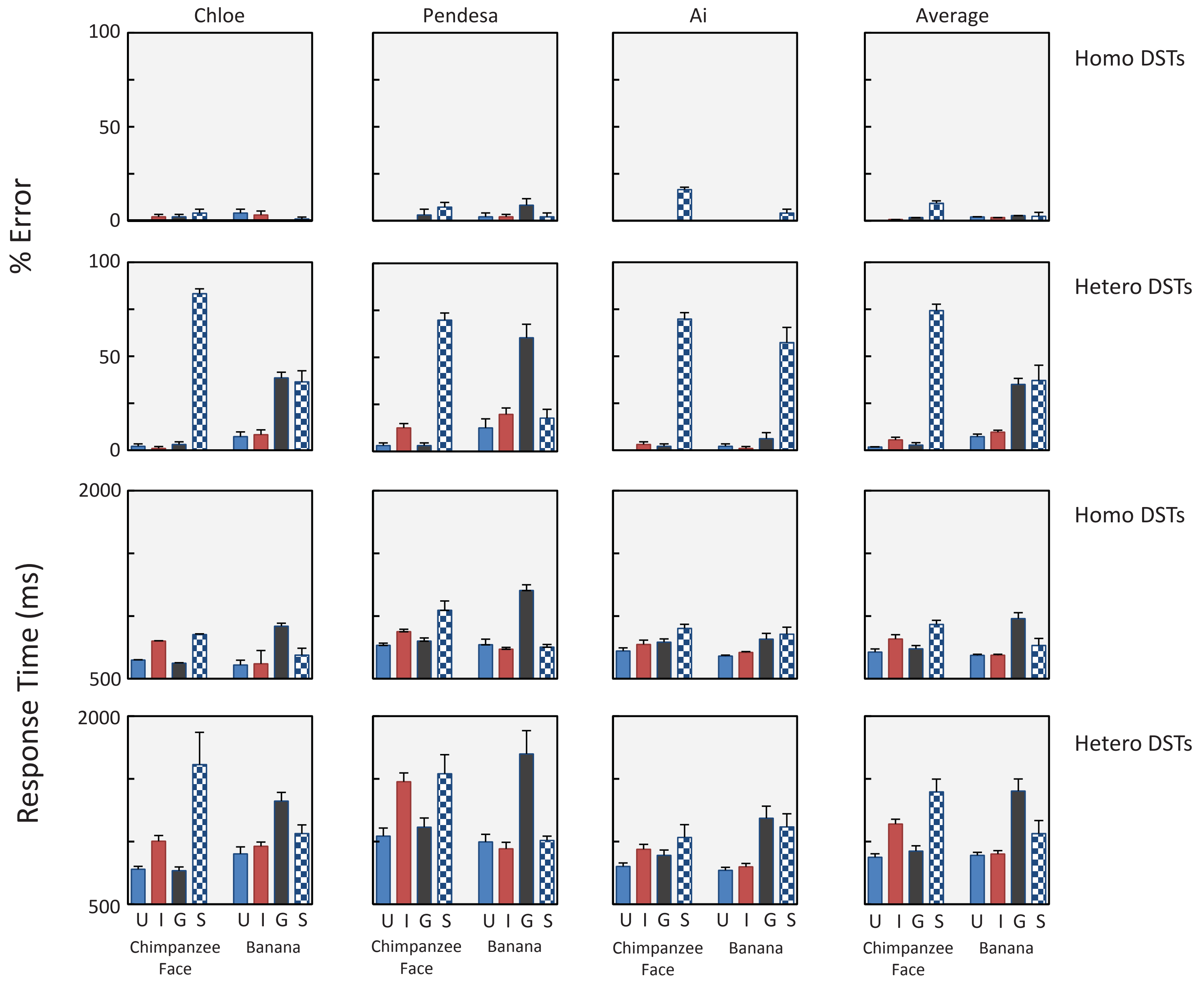

Figure S4. Individual and averaged data of Experiment 2. Upper panels: percentage of error trials for each target. Lower panels: response times on correct trials. Error bars show standard errors of mean across sessions for the individual data and those across chimpanzees for averaged data.
Homo DSTs = homogeneous-distractor trials, Hetero DSTs = heterogeneous-distractor trials. U: upright, I: inverted, G: grayscale, S: scrambled.

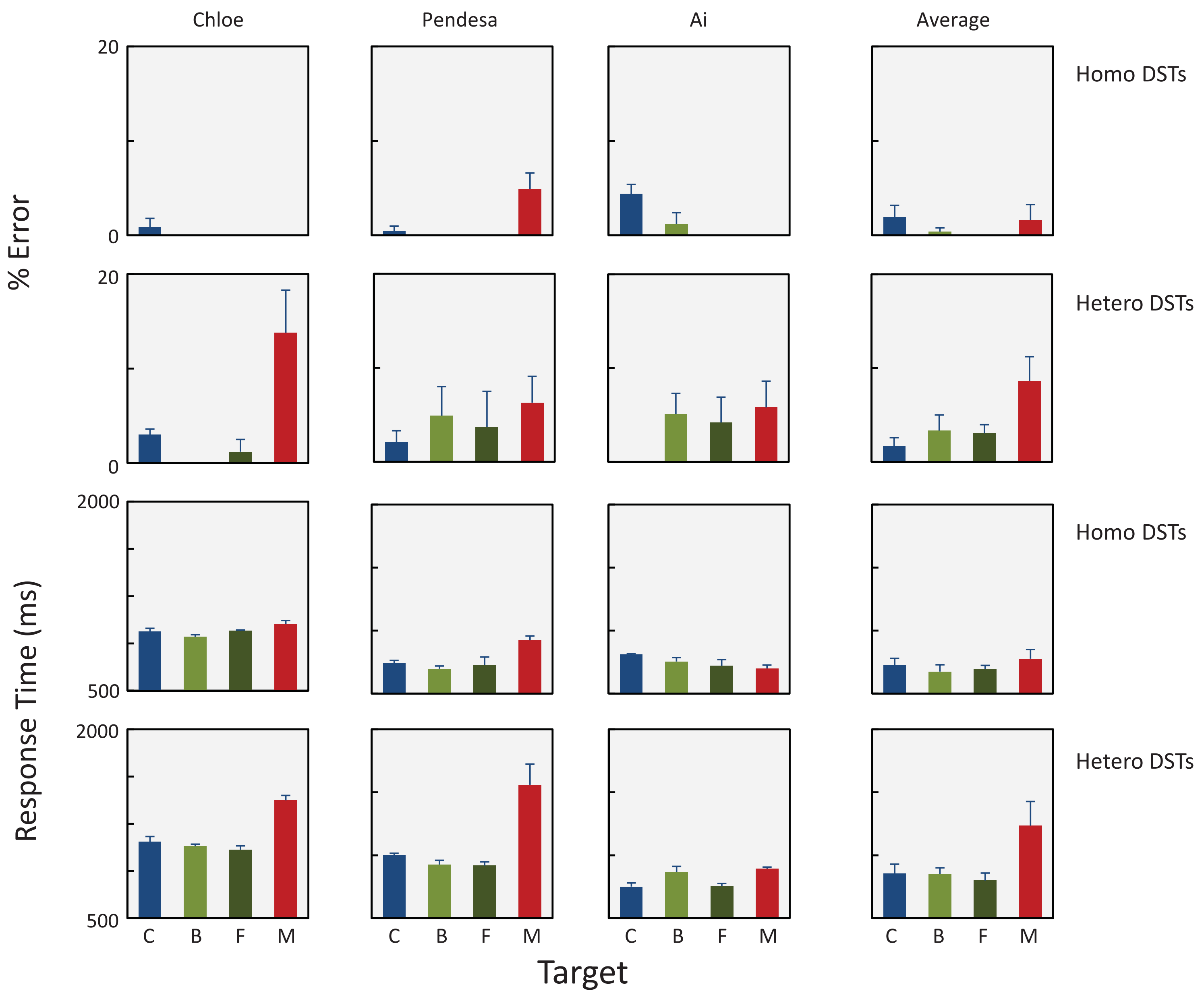

Figure S5. Individual and averaged data of Experiment 4. Upper panels: percentage of error trials for each target. Lower panels: response times on correct trials. Error bars show standard errors of mean across sessions for the individual data and those across chimpanzees for averaged data. Homo DSTs = homogeneous-distractor trials, Hetero DSTs = heterogeneous-distractor trials. C: chimpanzee face, B: human baby face, F: Human adult female face, M: Japanese monkey face.

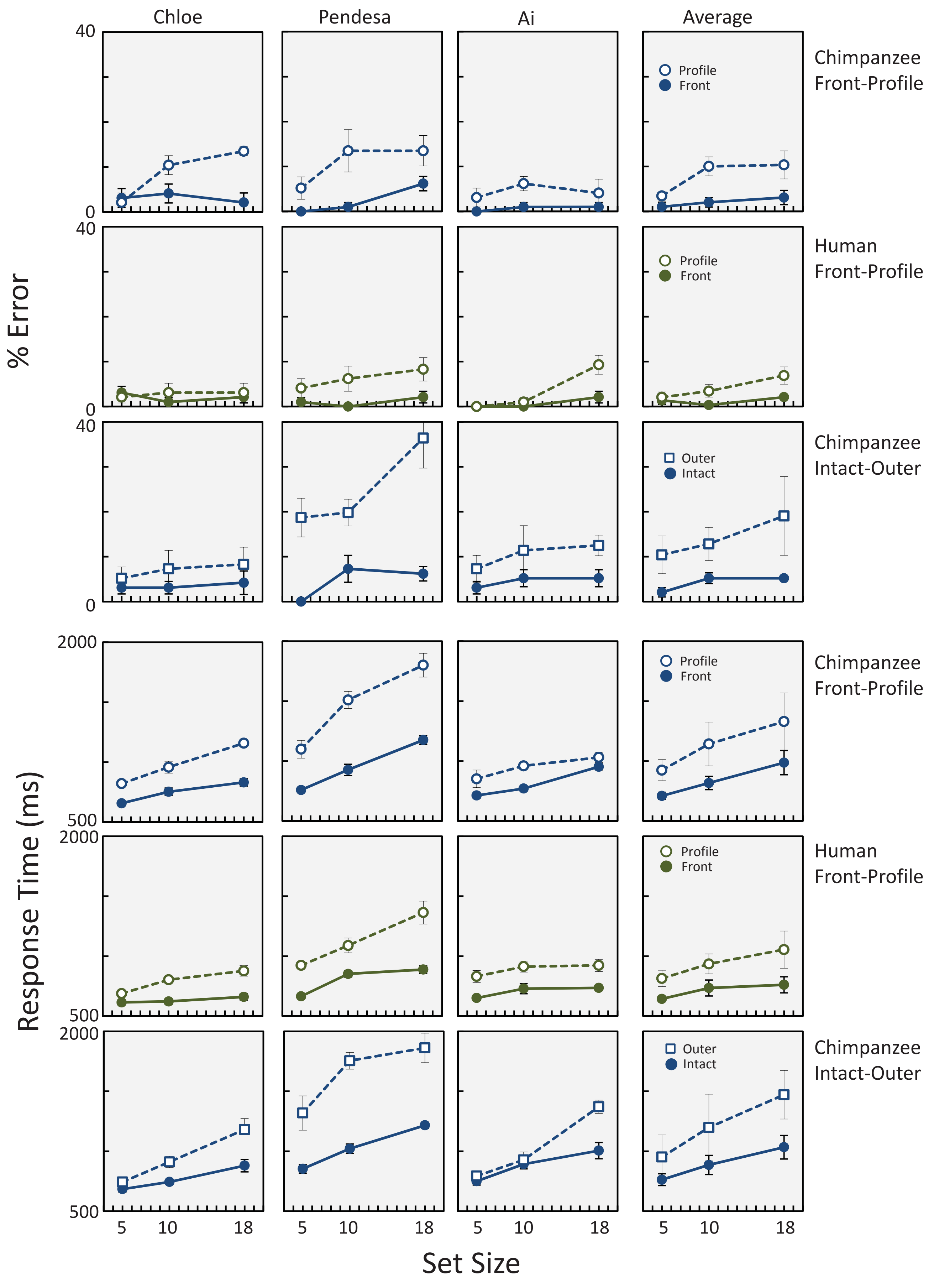

Chloe
Pendesa
Ai
Average
Chimpanzee Front-Profile
Human Front-Profile
Chimpanzee Intact-Outer
Profile
Front
Outer
Intact
% Error
Response Time (ms)
Set Size
40
0
2000
500
5
10
18

Figure S6. Individual and averaged data of Experiment 5. Upper panels: percentage of error trials for each target. Lower panels: response times on correct trials. Error bars show standard errors of mean across sessions for the individual data and those across chimpanzees for averaged data.
